# Hospital Costs of Intracranial Haemorrhage in Patients with Acute Pulmonary Embolism: Possible Implications for Emerging Therapies

**DOI:** 10.1055/a-2511-4599

**Published:** 2025-01-27

**Authors:** Konstantinos C. Christodoulou, Katharina Mohr, Timo Uphaus, Max Jägersberg, Luca Valerio, Ioannis T. Farmakis, Lukas Hobohm, Harald Binder, Stavros V. Konstantinides, Karsten Keller

**Affiliations:** 1 Center for Thrombosis and Hemostasis (CTH), University Medical Center of the Johannes Gutenberg-University, Mainz, Germany; 2 Institute of Medical Biometry and Statistics, Faculty of Medicine and Medical Center, University of Freiburg, Freiburg, Germany; 3 Department of Neurology, Focus Program Translational Neuroscience (FTN) and Immunotherapy (FZI), Rhine Main Neuroscience Network (rmn2), University Medical Center of the Johannes Gutenberg-University, Mainz, Germany; 4 Department of Neurosurgery, University Medical Center of the Johannes Gutenberg-University, Mainz, Germany; 5 Department of Cardiology I, University Medical Center of the Johannes Gutenberg-University, Mainz, Germany; 6 Department of Cardiology, Democritus University of Thrace, Alexandroupolis, Greece

Pulmonary embolism (PE) is a potentially life-threatening event, 1 with increasing incidence over the past decades. 2 3 Treatment of acute PE is currently undergoing profound change, and novel reperfusion treatment modalities rapidly entering the market need to show, among others, whether they can reduce the complications associated with systemic fibrinolysis and thus justify their elevated costs. 4 5 6 In this regard, intracranial haemorrhage (ICH) is in the focus of interest, 7 8 9 since it is associated with a high early case fatality and seriously impacts the patients' well-being and quality of life over the long term. We therefore examined the impact of ICH on reimbursed hospital costs (reflecting the payer's perspective) in patients with acute PE, and dissected the main cost drivers. Data from all patients hospitalized in Germany (with the exception of a very small minority admitted to military hospitals and psychiatric centres) with a main diagnosis of PE (ICD-10 code I26) during the period 2016–2020 (source: RDC of the Federal Statistical Office and the Statistical Offices of the Federal States, DRG Statistics 2016–2020; own calculations) were analyzed as previously described. 3 10 11 We stratified patients by the occurrence of ICH, identified by at least one of the following ICD-10 codes: I60, I61, I62, G95.10, S06.33, S06.5, S06.6. We analyzed all procedure codes related to the treatment of ICH, including surgical, endoscopic, or interventional drainage of intracranial haematomas (5-012.0, 5-012.2, 5-013.1, 5-013.4, 5-014.1, 5-022.0, 5-022.00, 5-022.01, 5-022.02, 5-022.0x, 5-022.20, 5-022.y, 5-033.2, 5-034.1, 5-034.4, 5-038.0, 8-020.d, and/or 8-151.1). Finally, we included multidisciplinary treatment in specialized facilities, coded as 8-981 or 8-98b. 12 13 The statistical software SPSS® for Windows, Version 20.0 (Armonk, NY: IBM Corp) was used for data analysis.

Overall, 283,280 patients (median age 71.0 [IQR 58.0–80.0] years; 51.4% women) were hospitalized in Germany due to acute PE in the above period. Median length of hospital stay was 7.0 (4.0–10.0) days; ICH was coded in 946 (0.3%) patients. More specifically, ICH occurred in 1.9% of the patients ( *n* = 12,907) who received systemic thrombolysis; 3.1% of those underwent surgical embolectomy and 6.1% of the patients treated with veno-arterial extracorporeal membrane oxygenation (VA-ECMO). PE patients suffering from ICH were older (74.0 [63.0–81.0] vs. 71.0 [58.0–80.0] years; *P* < 0.001) and slightly more frequently male (51.8%, vs. 48.5% of those without ICH; *P* = 0.046). ICH was associated with longer in-hospital stay (10.0 [3.0–17.0] vs. 7.0 [4.0–10.0] days; *P* < 0.001), more frequent admission to an intensive care unit (ICU; 47.8% vs. 12.7%; *P* < 0.001), and higher in-hospital case fatality (36.6% vs. 8.0%; *P* < 0.001).

The costs of hospitalization with PE complicated by ICH were approximately 2.8-fold higher than in its absence (mean: 10,805 ± 18,282 vs. 3,917 ± 4,952 euros; *P* < 0.001). Mean costs were highest in the year 2016 (13,062 ± 23,536 euros), decreasing progressively until 2020 (8,232 ± 13,598 euros). This trend was likely driven by adaptations in the hospital reimbursement system and possibly by more effective prevention of complications, notably pneumonia. 12 Moreover, the drop in costs during the first pandemic year 2020 probably resulted from a combination of medical and social factors as discussed previously. 10

Reimbursed hospitalization costs of PE with ICH varied with the patients' age: peak mean costs were detected in the second (37,685 ± 36,455 euros) and fifth (24,092.4 ± 33,639.6 euros) decades of life. Interestingly, PE patients ≤72 years had higher treatment costs than those 73 years or older (15,178 ± 23,396 and 7209 ± 11,444 euros, respectively; *P* < 0.001). This finding, supporting the results of a recent systematic review, 14 is likely due to (i) a higher early case fatality in older patients and (ii) more intensive diagnostic efforts and treatment escalation (for both PE and ICH) in younger patients.

Table 1 displays the main hospitalization cost drivers in patients with PE and ICH; the cut-off of 10,000 euros was chosen based on the fact that it best defines the reimbursement of intermediate- or high-risk PE receiving advanced treatment in the German DRG-based system. 15 Similar to what has been described for intracranial bleeding in the overall population, 10 16 17 18 comorbidity burden reflected by the Charlson comorbidity index (CCI) significantly contributed to hospitalization costs (CCI ≤3: 9,850 ± 16,169; CCI >3: 10,999 ± 18,684 euros; *P* = 0.041). Additionally, mean costs of PE complicated by ICH were higher in hospitals serving rural (11,313 ± 18,594 euros) compared to those in urban (10,555 ± 21,956 euros) and suburban (10,107 ± 14,664 euros) areas ( *P* < 0.001). Our results thus differ from those of US studies reporting that urban and teaching hospitals generated the highest costs in the treatment of haemorrhagic stroke 14 ; a possible explanation may be the longer hospital stay in rural hospitals and the more frequent drainage of intracranial haematomas in suburban hospitals in Germany (4.3%, compared to 3.2% in rural and 3.1% in city hospitals).

**Table 1:** Patient characteristics, treatment, and in-hospital course of patients with pulmonary embolism (PE) and intracranial haemorrhage (ICH) during the years 2016–2020 in Germany, stratified by hospitalization costs

Parameters	Costs ≤10,000 € ( *n* = 709; 74.9%)	Costs >10,000 € ( *n* = 237; 25.1%)	*P* -value
Age, *median (IQR)*	76.0 (67.0–82.0)	66.0 (54.5–76.0)	**<0.001**
Age ≥70 years	488 (68.8%)	96 (40.5%)	**<0.001**
Female sex	340 (48.0%)	116 (48.9%)	0.792
**Cardiovascular and VTE risk factors**			
Obesity	35 (4.9%)	26 (11.0%)	**0.001**
Diabetes mellitus	132 (18.6%)	48 (20.3%)	0.579
Arterial hypertension	346 (48.8%)	104 (43.9%)	0.189
Cancer	55 (7.8%)	28 (11.8%)	0.056
**Comorbidities**			
Coronary artery disease	92 (13.0%)	24 (10.1%)	0.247
Heart failure	221 (31.2%)	100 (42.2%)	**0.002**
Peripheral artery disease	15 (2.1%)	7 (3.0%)	0.459
Atrial fibrillation/flutter	140 (19.7%)	57 (24.1%)	0.158
Chronic obstructive pulmonary disease	43 (6.1%)	23 (9.7%)	0.057
Acute or chronic renal failure	178 (25.1%)	121 (51.1%)	**<0.001**
Chronic anaemia	38 (5.4%)	35 (14.8%)	**<0.001**
**Clinical severity**			
Intermediate- or high-risk PE	379 (53.5%)	179 (75.5%)	**<0.001**
RV dysfunction	343 (48.4%)	157 (66.2%)	**<0.001**
Haemodynamic instability	135 (19.0%)	116 (48.9%)	**<0.001**
**Treatment**			
Admission to intensive care unit	246 (34.7%)	206 (86.9%)	**<0.001**
‘Complex medical treatment’	12 (1.7%)	11 (4.6%)	**0.011**
Drainage of intracranial haematoma	4 (0.6%)	29 (12.2%)	**<0.001**
Systemic thrombolysis for PE	157 (22.1%)	82 (34.6%)	**<0.001**
Catheter-directed treatment for PE	20 (2.8%)	8 (3.4%)	0.660
**Length of stay**			
Length of in-hospital stay (days)	7.0 (2.0–13.0)	20.0 (12.0–32.0)	**<0.001**
**In-hospital outcomes**			
In-hospital mortality	240 (33.9%)	106 (44.7%)	**0.003**
Ischaemic stroke	44 (6.2%)	43 (18.1%)	**<0.001**
Pneumonia	189 (26.7%)	122 (51.5%)	**<0.001**
Acute renal failure	75 (10.6%)	100 (42.2%)	**<0.001**
Transfusion of blood constituents	57 (8.0%)	117 (49.4%)	**<0.001**

Abbreviations: IQR, interquartile range; PE, pulmonary embolism; RV, right ventricle/ventricular; VTE, venous thromboembolism.

Note: The term ‘complex medical treatment’ denotes treatment in a multidisciplinary stroke unit. *P* values lower than 0.05 (two-sided) were considered to be statistically significant and are set in bold.

In our study, the impact of ICH on the hospitalization costs for PE was smaller than might be expected; this can be explained, at least in part, by the high early case fatality of PE complicated by ICH. In addition, considering the low overall incidence of ICH in PE (0.3%), the added hospitalization costs imposed by this complication might appear rather moderate in the German healthcare system. However, this estimate is only a part of the entire picture due to (i) the substantial burden imposed by premature loss of life on the deceased patient's family and the society, and (ii) the fact that survivors of PE complicated by ICH continue to suffer from a broad range of symptoms often causing severe chronic disability and recurrent hospitalizations in addition to indirect costs. 19 20 21 Haemorrhagic stroke has been associated with a high disease burden as measured by disability-adjusted life-years, 22 and the economic burden of stroke due to loss of productivity was estimated at 12 billion euros in European countries in the year 2017. 23 In the present study, it was not possible to analyze the post-discharge costs of PE complicated by ICH, since these are not linked to the German nationwide hospitalized population. A further potential limitation is that our results are based on ICD-10 and procedure codes; underreporting (or overreporting) of cases or treatments cannot be excluded. 24 25 26 Nevertheless, the coding performed by German hospitals undergoes regular quality controls to ensure correct and transparent reimbursement.

The present analysis highlights the (socio-)economic impact of ICH associated with acute PE in a national healthcare system. Our results may prove helpful for evaluating, apart from the clinical benefits, the potential cost savings of new interventional modalities for the treatment of acute PE. If ongoing randomized trials confirm the potential of these procedures to lower, among others, the risk of life-threatening or incapacitating ICH, the emerging technological advances in reperfusion treatment may help to reduce the overall disease burden of PE, in the acute phase and over the long term.
